# A232 CAN WE ACCURATELY PREDICT SEVERE ACUTE PANCREATITIS IN CHILDREN? A RETROSPECTIVE REAL-LIFE COHORT OF HOSPITALIZED PATIENTS

**DOI:** 10.1093/jcag/gwae059.232

**Published:** 2025-02-10

**Authors:** C Korman, D Elhaoua, S Blain, T Li, M Mohamed, A David, C Deslandres, U Halac, M Dirks, K Grzywacz, M Lallier, F Alvarez, P Jantchou

**Affiliations:** McGill University Faculty of Medicine and Health Sciences, Montreal, QC, Canada; Universite de Montreal Faculte de Medecine, Montreal, QC, Canada; Centre Hospitalier Universitaire Sainte-Justine Division of Gastroenterology Hepatology and Nutrition, Montreal, QC, Canada; Centre Hospitalier de l’Universite de Montreal, Montreal, QC, Canada; Universite de Montreal Faculte de Medecine, Montreal, QC, Canada; Centre Hospitalier de l’Universite de Montreal, Montreal, QC, Canada; Centre Hospitalier Universitaire Sainte-Justine Division of Gastroenterology Hepatology and Nutrition, Montreal, QC, Canada; Centre Hospitalier Universitaire Sainte-Justine Division of Gastroenterology Hepatology and Nutrition, Montreal, QC, Canada; Centre Hospitalier Universitaire Sainte-Justine Division of Gastroenterology Hepatology and Nutrition, Montreal, QC, Canada; Centre Hospitalier Universitaire Sainte-Justine Division of Gastroenterology Hepatology and Nutrition, Montreal, QC, Canada; Centre Hospitalier Universitaire Sainte-Justine Division of Gastroenterology Hepatology and Nutrition, Montreal, QC, Canada; Centre Hospitalier Universitaire Sainte-Justine Division of Gastroenterology Hepatology and Nutrition, Montreal, QC, Canada; Centre Hospitalier Universitaire Sainte-Justine Division of Gastroenterology Hepatology and Nutrition, Montreal, QC, Canada

## Abstract

**Background:**

Severe acute pancreatitis (SAP) affects nearly a quarter of pediatric patients with acute pancreatitis (AP). However, previous studies suggested that severity scores used in adults have low sensitivity for predicting pediatric SAP. Uncertainty remains regarding the factors associated with SAP in children.

**Aims:**

The primary aim was to validate SAP predictive scores in a Canadian pediatric population and identify associated predictive factors.

**Methods:**

A retrospective observational cohort study was conducted in a tertiary hospital in Montreal, Canada (January 2014-December 2021). SAP was defined by intensive care unit (ICU) admission and/or complications including but not limited to fluid collection, necrosis, and pseudocyst. Firstly, multivariate logistic regression analyses were performed to evaluate the relationship between clinical and laboratory factors and the outcome (SAP). A predictive model was also developed based on age, co-existence of chronic disease (e.g., neoplasia, obesity), C-reactive protein (CRP) levels within 48 hours of admission, baseline urea levels, and sex. Secondly, receiver operator characteristic (ROC) curves were plotted for various published scores (Balthazar (n=55), DeBanto (n=118), Glasgow modified (n=38), Ranson (n=20)) and our model (n=122).

**Results:**

Among 208 patients (51.0% male, median (interquartile range (IQR)) age [12.5 (7.1;15.3) years]), 76 (36.5%) developed SAP. Of these, 14 (6.7%) were admitted to the ICU, and 68 (32.7%) developed complications. The median (IQR) hospital duration was 5.0 (2.5;11.0) days. The ROC for Balthazar, DeBanto, Glasgow modified, Ranson, and our model showed an area under the curve of 0.63, 0.58, 0.59, 0.58, and 0.80, respectively. Balthazar demonstrated the highest specificity (100%). Ranson showed the highest sensitivity (50%). Our model demonstrated a specificity of 78% and a sensitivity of 58%. Each unit increase in CRP within 48 hours of admission and each unit increase in urea at admission were associated with a higher risk of SAP (OR_CRP_=1.02, 95% CI: 1.01-1.03; OR_urea_=1.32, 95% CI: 1.08-1.61) whereas each unit increase in lipase within 48 hours of admission (minimum 3.0 U/L; maximum 5745.0 U/L) were not predictive of SAP.

**Conclusions:**

Existing scores display low predictive value for SAP. Our new model, which includes age, CRP and urea levels, sex, and the presence of co-existing conditions, demonstrates good predictive value for pediatric SAP and warrants external validation in larger pediatric cohorts.

**Funding Agencies:**

None

